# A Comparison of pical Root Resorption in Incisors after Fixed Orthodontic Treatment with Standard Edgewise and Straight Wire (MBT) Method

**Published:** 2013-09

**Authors:** SM Zahed Zahedani, M Oshagh, Sh Momeni Danaei, SMM Roeinpeikar

**Affiliations:** a Postgraduate Student of Oral & Maxillofacial Medicine, Dept. Oral & Maxillofacial Medicine, School of Dentistry, Shiraz University of Medical Sciences, Shiraz, Iran.; b Orthodontics Research Center, Dept. of Orthodontics, School of Dentistry, Shiraz University of Medical Sciences, Shiraz, Iran.

**Keywords:** Root Resorption, Standard Edgewise, Straight Wire (MBT)

## Abstract

**Statement of Problem:** One of the major outcomes of orthodontic treatment is the apical root resorption of teeth moved during the treatment. Identifying the possible risk factors, are necessary for every orthodontist.

**Purpose:** The aim of this study was to compare the rate of apical root resorption after fixed orthodontic treatment with standard edgewise and straight wire (MBT) method, and also to evaluate other factors effecting the rate of root resorption in orthodontic treatments.

**Materials and Method:** In this study, parallel periapical radiographs of 127 patients imaging a total of 737 individual teeth, were collected. A total of 76 patients were treated by standard edgewise and 51 patients by straight wire method. The periapical radiographs were scanned and then the percentage of root resorption was calculated by Photoshop software. The data were analyzed by Paired-Samples t-test and the Generalized Linear Model adopting the SPSS 15.0.

**Results:** In patients treated with straight wire method (MBT), mean root resorption was 18.26% compared to 14.82% in patients treated with standard edgewise technique (*p*< .05). Male patients had higher rate of root resorption,statistically significant (*p*< .05). Age at onset of treatment, duration of treatment, type of dental occlusion, premolar extractions and the use of intermaxillary elastics had no significant effect on the root resorption in this study.

**Conclusion:** Having more root resorption in the straight wire method and less in the standard edgewise technique can be attributed to more root movement in pre-adjusted MBT technique due to the brackets employed in this method.

## Introduction

External root resorption was first described by Bates in 1856 in a paper titled “Absorption” [1], and later in 1914, Ottolengui related this damage to orthodontic treatments [2]. 

Throughout the years, extensive orthodontic treatments are recognized as a major risk factor for increasing the prevalence and severity of root resorption. Specially heavy forces are most damaging [3]. Many studies are conducted to find the magnitude of force resulting in root resorption of teeth. But, roots are three dimensional and studying root resorption with two dimensional radiographs are difficult [4]. 

The most common affected teeth are the maxillary incisors followed by the mandibular incisors; specially the ones with abnormal root shape [5-8]. 

There are many possible risk factors that can be associated with this condition. These factors can be divided into two groups of pre-treatment and treatment factors.

The pre-treatment factors mentioned in some studies which make the patient prone to root resorption are: root resorption existing before the start of treatment [9], tongue thrust, finger sucking and nail biting habits [5, 10], genetic susceptibility, gender, use of medication, existing overjet [11], history of trauma, impacted maxillary canines, number of missing teeth [7], asthmatic patients [8] and patients starting treatment at an older age [9, 12]. Although in other studies asthmatic patients [7], age at start of treatment [13], gender [9, 12], overjet and overbite [12] are not recognized as risk factors for root resorption in orthodontic treatments. Recently the effect of history of trauma to teeth and root morphology are questioned as possible risk factors [3].

Several factors can initiate or progress root resorption during the treatment. Several investigators have suggested that longer active treatment time results in greater resorption [9, 14], even though other studies found no relation between duration of treatment and root resorption [11]. Duration of treatment with rectangular arch wires, intermaxillary elastics [5] and first premolar extractions [9] are also found as a risk factor for root resorption. 

Another factor that is assessed in some studies is the effect of different appliances and different techniques used to treat orthodontic patients [12-16]. 

In 1928 Angle introduced one of the most common fixed orthodontic appliances used today; the standard edgewise technique [17]. The name “Edgewise” was used because of the change in bracket slots from vertical to horizontal and also placement of a rectangular wire in the slot. But still faciolingual bends (first order bends) in the archwires were needed in this technique for different teeth anatomy [17]. 

In the 1970s, the “straight wire” appliance was developed by Andrews [18]. In this method the base of the bracket was designed in a special way for each individual tooth, thus minimizing the number of bends needed in the archwires [18]. 

The MBT bracket system is named after the designers; Mclaughlin, Bennett and Trevisi [19]. It is a pre-ajusted bracket systems made for use with light, continuous forces, lacebacks and bendbacks, and it also works well with sliding mechanics [19]. 

The effect of straight wire orthodontic treatments on root resorption has been evaluated in other studies [13-14, 16]. But the MBT bracket system has not been compared to the standard edgewise treatment yet. This technique is becoming popular among general practitioners in Iran because of the pre-adjusted brackets and their ease of use.

The main objective of this study is to compare the percentage of root resorption in maxillary and mandibular incisors after orthodontic fixed technique standard edgewise (SEW) (0.022 inch slot), and the straight-wire appliance (MBT) technique (0.022 inch slot). Also in this study we evaluate the effect of pre-treatment factors such as, age at the start of treatment, gender, dental occlusion and also treatment factors such as duration of treatment, use of intermaxillary elastics and premolar extractions, on the rate of root resorption in incisors.

## Materials and Method

In this non-concurrent retrospective cohort study, longitudinal database were gathered from existing documents of patients treated in two private offices in Shiraz, Iran. The sample was chosen from all Iranian patients who had been treated from the year 1998 to 2005 in these offices. From the 600 files studied (140 MBT and 460 SEW patients), 127 patients, 31 male and 96 female, with the age range of 9-25 (mean 14.77 ±0.376) were chosen. 76 patients were treated with SEW method and 51 patients had MBT treatment.

The inclusion criteria were:

1. Iranian patients who had finished active treatment with either standard edgewise technique (with 0.022 inch slot) or straight wire technique (MBT with 0.022 inch slot)
2. Existence of good resolution periapical radiographs of the incisors taken with the long cone paralleling technique using Rinn XCP (Troply 94 Vincennes, Minorex, France) before and after active treatment
3. Patients with healthy periodontal tissues
4. Patients who had incisors with closed apices

The exclusion criteria were:

1. Patients that presented root resorption at the pretreatment stage
2. Orthodontic retreatment cases
3. Patients presenting a history of genetic or developmental abnormalities or hormonal imbalance
4. History of oral habits
5. Incisors with the history of RCT, trauma and/or attrition
6. Existence of impacted canines
7. Rotation of incisors before the start of treatment
8. Incisors with interproximal reduction
9. Incisors with abnormal root shapes such as bottle shape, cone shape and dilacerated roots
10. History of oral surgery
11. Patients who underwent premolar extraction only in one jaw or one quadrant
12. Patients with different right and left dental occlusions (Angle’s occlusion)

All this information was gained from the records, radiography and photography of the patients.

The 76 patients treated with the standard edgewise technique, had 0.022-inch twin standard brackets (3M Unitek, Monrovia, California), with 0 degree torque and 0 degree angulation. The usual wire sequence began with a 0.017 co-axial, followed by 0.014, 0.016, 0.018 inch stainless steel wire (3M Unitek, Monrovia, California). 

The 51 patients treated with the Straight Wire System (MBT) had 0.022-inch twin straight wire edgewise brackets (Dentarum GmbH & Co. KG; Germany). In this system the built-in characteristics were: for the central incisors, torque +22 degrees, angulations +5 degrees, base height 0.79mm; and for the lateral incisors, torque +14 degrees, angulations +9 degrees, base height 1.28 mm.

The wire sequence and mechanics of the MBT group were similar to the Simplified Standard Edgewise Technique, beginning with a 0.175 co-axial or 0.016 nitinol wire (3M unitek, Monrovia, California), followed by 0.014, 0.016, 0.018, 0.016×0.022, 0.017× 0.025 and finally a 0.019 × 0.025 inch stainless steel wire (3M Unitek, Monrovia, California). 

To quantify resorption in the two groups, 1948 pre- and post- treatment periapical radiographs of the maxillary and mandibular incisors, were gathered from the records. After eliminating the poor projected radiographs, 737 teeth were evaluated, 416 teeth from patients treated by the standard edgewise technique (76 patients) and 321 teeth from patients treated by the MBT method (51 patients).

The radiographs were scanned with Microtek i800 scanner (Microtek International, Science-Based Industrial Park, Hsinchu, Taiwan) (dpi=300), a special scanner used for negative films, and measured by the use of Photoshop S3 software.

To calculate the percentage of root resorption, the length of the teeth before and after treatment (L1, L2) were measured from the mid-point of the incisal edge to the apex. All measurements were obtained by projecting these points as accurately as possible along the root canal as the long axis of the teeth. 

L1= Root length before active treatment

L2= Root length after active treatment

**Figure 1:**
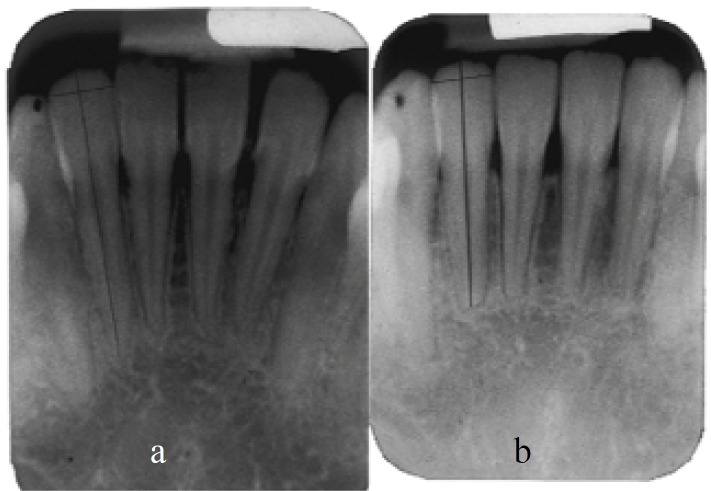
Calculating the root resorption percentage in a lower mandibular lateral incisor treated with the MBT method a Root length and greatest mesiodistal width, before the start of treatment b Root length and greatest mesiodistal width of the same tooth after treatment.

Also for correcting magnification in the radiographs, a fixed measurement which was assumed to be unchanged over the observation period, was used. This fixed measurement was the mesiodistal width of the crown, which was measured on the radiographs before (md1) and after (md2) treatment by connecting the points on the mesial and distal edge of incisal level (figure 1). 

md1= Mesiodistal width before treatment

md2= Mesiodistal width after treatment

If the mesiodistal width was different in the pre- and post-radiographs, the length of the tooth before and after treatment was divided by the mesiodistal width. 

n_1_= L_1_/md_1 _n_2_= L_2_/md_2_

And then the difference between the measurements before and after treatment was calculated, and root resorption was expressed as a percentage shortening per tooth: (n_1_-n_2_)/n_1_× 100 = PR

RR= the percent of tooth shortening (root resorption) 

All measurements were performed by one examiner, who did not know the patients’ names, descriptions and the methods of treatment used for each patient. The accuracy of the measurements was assessed by analyzing the difference between measurements on 50 radiographs of the incisors from seven randomly selected patients measured again by the same examiner 3 weeks after the first measurements. This re-evaluation showed that in 90.8 % of the cases the measurements were the same.

The statistical test used for analyzing the mandibular versus maxillary, and lateral versus central incisors was the paired-samples t-test. Also for evaluating the differences between the treatment duration of maxilla and mandible for each patient the paired sample t-test was used. For evaluating the effect of pre-treatment and treatment factors, and also the method of treatment, the Generalized Linear Model was used. 

## Results

The descriptive analysis are shown in table 1 and 2. 

**Table 1:** Frequency of the factors evaluated

**Factrors Evaluated**		**N**	**Percentage %**
Type of treatment	SEW	76	59.8
	MBT	51	40.2
Sex	Male	31	24.4
	Female	96	75.6
Premolar extractions		65	51.2
Intermaxillary elastics		90	70.9
Dental occlusions	I	68	53.5
	II	45	35.4
	III	14	11.0

N= number of patients

The differences in the treatment duration of the upper jaw compared to the lower jaw for each patient, evaluated with the paired sample t-test, were not significant (*p*> 0.05). So the mean duration time of treatment of both jaws was chosen as the treatment duration for each patient. 

Data analysis showed that the mean root resorption for all measured teeth was 16.21% of the tooth length before the start of treatment (Table 3). Also the lower centrals had the least amount of root resorption between all the groups of teeth measured (14.89%). The lower laterals were next and the upper centrals and laterals had almost the same amount of resorption. The comparison of these percentages were not significantly different for each patient (*p*> 0.05), so an average value of all the teeth measured for each patient was used as the dependent variable of the patient in the generalized linear model. 

In patients treated with straight wire method (MBT) mean root resorption for all teeth was 18.26% compared to 14.82% in patients treated with standard edgewise technique (*p*< .05).

The results of the generalized linear model is summerized in table 4. On the basis of this test the root resoprtion in patients treated with the MBT method was significaltly more than the SEW group (*p*= 0.002).

Of the other factors evaluated, only male gender compared to female had significant effect on the rate of root resorption (*p*< .05). We found no association betw-een age, different dental occlusions (Angle’s ooclusions), treatment duration, using intermaxillary elastics, premo-lar extraction and the rate of root resorption (*p*> .05).

## Discussion

In this study the incisors were chosen for the comparison of root resorption in two orthodontic techniques, be-cause it has been reported that the maxillary incisors are the most susceptible teeth to root resorption and after that the mandibular incisors show the most resorption [5-8].

**Table 2:** Treatment duration in each group (MBT and SEW)

**Method of Treatment**	**Range of Treatment Duration**		**Mean Treatment Duration**	
	**Mandible**	**Maxilla**	**Mandible**	**Maxilla**
SEW	11-45 months	12-45 months	26.85 months	26.93 months
MBT	7-33 months	9-33 months	18.71 months	19.81 months
Total	7-45 months	9-40 months	23.75 months	23.98 months

**Table 3:** Mean percentage of root resorption in the teeth measured

**Treatment Method**	**All teeth**		**Maxillary centrals**		**Maxillary laterals**		**Mandibular centrals**		**Mandibular laterals**	
	**N**	**%**	**N**	**%**	**N**	**%**	**N**	**%**	**N**	**%**
SEW	416	14.82	107	15.28	98	15.42	117	13.12	94	13.91
MBT	321	18.26	81	18.77	89	18.92	77	17.74	74	18.98
Total	737	16.21	188	16.76	187	16.99	194	14.89	168	16.05

N= number of Teeth measured                              % =mean percentage of root resorption calculated

**Table 4:** Results of the Generalized Linear Model Test

**Parameter**		**Coefficient Value**	**P**
Type of treatment	(SWE)	-.044	.002
	(MBT)	0*	.
Sex	Male	.029	.032
	Female	0*	.
Premolar extraction		.0334	.163
Intermaxillary elastics		.014	.301
Dental Occlusion	Class I	-.028	.174
	Class II	-.024	.245
	Class III	0*	.
Age		.001	.507
Treatment duration		.000	0.628

*Set to zero because this parameter is redundant

Anatomic variability of these teeth can be a possibility for this difference [12].

Different methods for evaluating root resorption exist. Panoramic films have been used because they are easy to obtain and the patient is less exposed [19], but unfortunately the shape of the root seen in this radiography, is less accurate, especially for the anterior teeth. The amount of root resorption will also be overestimated [20]. The periapical radiographs are reported as the best method to evaluate root resorption because of less image distortion [21]. With the introduction of CBCT technology into clinical orthodontics, it is recommended that root resorption be evaluated using CBCT images which not only improves accuracy but also provides three-dimensional evaluations [22]. 

In this study, we used standard periapical radiographs with the long-cone paralleling technique and image distortion between the pre- and post- treatment radiographs was corrected using crown dimensions. This method was originally introduced by Linge and Linge [23]. All radiographs of the patients being studied were taken in one radiology center and measured by only one observer. The measurements in this study were obtained by the use of Photoshop software which enables better magnification of the radiographic images for a more precise measurement of the points. 

Although magnifications in parallel periapical radiographs are the same in horizontal and vertical dimensions, calculating the mesiodistal width or correcting magnification might be more reliable than the use of crown length which was used in other studies [17, 24-25]. The crown length was measured by the use of the cementoenamel junction (CEJ) which is not detected accurately on radiographs. A percentage value is a better comparative value, since the differences in the root lengths of various teeth in millimeters make comparisons of root resorption values less meaningful [15-16]. But the short come of using a percentage is in shorter teeth, which might not have much resorption in millimeters but show great percentage of root resorption.

Negative values for root resorption indicating an increase in root length, was also seen in few measurements. This has also been previously reported and considering the age range of the sample, can be attributed to a real increase in root length [16] or to method error registering the apex [17, 26-27].

The main finding of this study was that root resorption after MBT orthodontic treatment was significantly more than standard edgewise treatment (*p*< 0.05). This finding may be attributed to inadvertent movement of the teeth in MBT technique because of bracket’s prescriptions. Also in the straight wire appliance more root movement is seen in the beginning of treatment [18]. The standard edgewise appliance completes root movement in the last (3rd) stage of treatment using wire bending [15]. This can lead to more root resorption in the straight wire method [28]. Duration of treatment was shorter in MBT method as can be seen in table 2. So for gaining ideal positions of teeth, root movements were faster, thus more force could have been used for this aim. This is in agreement with other studies which found more root resorption in straight wire techniques [16, 29]. But Mavragani et al. found that there was significantly more apical root resorption of both central incisors in the standard edgewise group than the straight-wire group. This difference may be related to the fact that their sample consisted of patients with class II division 1 malocclusions and the straight wire appliance used by them was different from the MBT appliance [14]. Also Santos et al. found that root resorption in patients treated with straight- wire system and nickel-titanium alloy was less than in patients treated by standard edgewise technique and stainless steel wires. The difference seen in Santos et al.’s results compared to the current study may be attributed to the different wire used by them (combination use of nickel- titanium wires in the straight wire technique and stainless- steel 

wires in the standard technique) [15]. 

In Janson et al.’s study it is also shown that different techniques of treatment have different outcomes of root resorption [13]. This is not in agreement with other studies which found no significant difference between different techniques [10, 12]. Also a comparison between the different techniques of Begg, Tweed standard edgewise and Roth straight wire, failed to show a technique that produces either more or less root resorption [20]. Weltman et al. reported that orthodontically induced inflammatory root resorption is unaffected by archwire sequencing and bracket prescription [13]. 

The result of samples consisting of patients treated by various professionals, can be less accurate [13]. In many studies it is concluded that the work of different clinicians has no significant effect on the results of an evaluation [5, 10, 13]. In this study having no more than two clinicians has reduced the effect of this bias. But nevertheless, it is better to have a sample treated by one clinician. 

In our study root resorption in males was significantly more than in females. This is in agreement with Sameshima et al.’s study [30]. Also in Nigul et al. study males had more root resorption than females but the differences were not statistically significant [31]. Some researchers have registered no difference in root resorption in each gender [9, 12, 26]. Ravanmehr et al. reported that although different levels of sexual hormones may be attributed to susceptibility to root resorption, but no difference is seen in males and females after treatment [26]. In Mohandesan et al.’s report the maxillary incisors of female patients showed more resorption than those of male patients but the effect of gender was found only for the maxillary lateral incisors [29]. Although it should be noted that in our study only 24.4% of the patients were male and 75.6% were female. This unequal distribution of gender might affect the results.

Even though not significantly different, in the sample studied by us it was observed that the older the age at start of treatment, the greater the amount of root resorption. This is in agreement with the studies of Nigul and Jagomagi [31], Mavragani et al. [14] and Jiang et al. [9]. It is reported that with increasing age, the areas of hyalinization and the duration of hyalinization increase but the ability to repair decreases [27, 29]. But Bishara et al. performed an extensive radiographic survey and found no systematic difference in root shortening between early and mid adulthood [8]. Also Mirabella et al. found no difference in root resorption of the teeth of adults compared to children [27]. 

In this study there was no significant difference in root resorption between extraction and non-extraction groups. It is in agreement with Nigul’s study which indicated that extraction treatment was not associated with excessive root resorption [31]. The result of the current study is not in agreement with other studies which described more resorption after extraction [9-11, 29]. 

This study also showed that patients who used intermaxillary elastics had no significant root resorption than others. It is in accordance to studies which found no relationship between treatment with inter-arch elastics and root resorption [10]. It is not in agreement with some studies which emphasized the risks that intermaxillary elastics could have on root resorption [5, 27]. Wearing elastics depends on patient cooperation and it must be stated that the treatment time which is reported in papers may not always reflect the real wearing time.

We found the same result of other studies which reported no significant relation between treatment duration and root resorption [12]. It is not in agreement with many studies which supported the significance of treatment duration in root resorption [9-10, 27, 29]. However treatment duration should not be considered the main factor for root resorption [32]. Perhaps the amount of tooth movement is the most important factor, and it is independent of treatment time. In some cases the appliance can be present with reduced action on the teeth, and in other cases, patients can frequently miss an appointment. Also treatment can be delayed because of professional preference in prolonging the intervals between activations [27]. Weltman et al. mentioned that 2-3 months stop in treatment can reduce the amount of root resorption [3]. 

Different dental occlusions presented no different rate of root resorption in our review. Vonder Ahe also reported the same amount of root resorption in class I and class II patients [33]. Other studies found that different dental occlusions show different amounts of root resorption [24, 34]. Salehi et al. reported that class II patients present with increase root resorption during orthodontic treatments [24]. But root resorption in Taner et al.’s report was on average 1mm for class I patients, 2mm for class II patients and also the central maxillary incisors had more root resorption in the class II group compared to class I patients in their study [34]. According to table 1, the sample size in our study was different in each occlusion group. Therefore, future evaluation must be conducted regarding dental occlusions and root resorption with more accurate sample sizes.

## Conclusion

Root resorption after orthodontic treatment can be affected by many local and systemic risk factors. One local factor is the technique of treatment used. In this study, root resorption after MBT orthodontic treatment was significantly more than standard edgewise orthodontic treatment. Orthodontists must keep in mind that different fixed methods used in orthodontic treatment, can affect the rate of root resorption in incisors. So in susceptible patients the treatment method must be chosen with careful concerns. 

## References

[1] Bates S (1856). Absorption. Br J Dent Science.

[2] Ottolengui R (1914). The physiological and pathological resorption of tooth roots. Items Interest.

[3] Weltman B, Vig KW, Fields HW, Shanker S, Kaizar EE (2010). Root resorption associated with orthodontic tooth movement: a systematic review. Am J Orthod Dentofacial Orthop.

[4] Chan EK, Darendeliler MA, Petocz P, Jones AS (2004). A new method for volumetric measurement of orthodontically induced root resorption craters. Eur J Oral Sci.

[5] Newman WG (1975). Possible etiologic factors in external root resorption. Am J Orthod.

[6] McNab S, Battistutta D, Taverne A, Symons AL (1999). External apical root resorption of posterior teeth in asthmatics after orthodontic treatment. Am J Orthod Dentofacial Orthop.

[7] Owman-Moll P, Kurol J (2000). Root resorption after orthodontic treatment in high- and low-risk patients: analysis of allergy as a possible predisposing factor. Eur J Orthod.

[8] Bishara SE, Vonwald L, Jakobsen JR (1999). Changes in root length from early to mid-adulthood: resorption or apposition?. Am J Orthod Dentofacial Orthop.

[9] Jiang RP, McDonald JP, Fu MK (2010). Root resorption before and after orthodontic treatment: a clinical study of contributory factors. Eur J Orthod.

[10] Sameshima GT, Sinclair PM (2001). Predicting and preventing root resorption: Part II Treatment factors. Am J Orthod Dentofacial Orthop.

[11] Sameshima GT, Sinclair PM (2004). Characteristics of patients with severe root resorption. Orthod Craniofac Res.

[12] Beck BW, Harris EF (1994). Apical root resorption in orthodontically treated subjects: analysis of edgewise and light wire mechanics. Am J Orthod Dentofacial Orthop.

[13] Janson GR, De Luca Canto G, Martins DR, Henriques JF, De Freitas MR (2000). A radiographic comparison of apical root resorption after orthodontic treatment with 3 different fixed appliance techniques. Am J Orthod Dentofacial Orthop.

[14] Mavragani M, Vergari A, Selliseth NJ, Bøe OE, Wisth PL (2000). A radiographic comparison of apical root resorption after orthodontic treatment with a standard edgewise and a straight-wire edgewise technique. Eur J Orthod.

[15] Santos ECA, Lara TS, Arantes FM, Coclete GA, Silva RS (2007). Computer-assisted radiographic evaluation of apical root resorption following orthodontic treatment with two different fixed appliance techniques. Rev Dent Press Ortodon Ortop Facial.

[16] Reukers EA, Sanderink GC, Kuijpers-Jagtman AM, van't Hof MA (1998). Radiographic evaluation of apical root resorption with 2 different types of edgewise appliances Results of a randomized clinical trial. J Orofac Orthop.

[17] Angel EH (1928). The latest and best in orthodontic mechanisms. Dent Cosmos.

[18] Andrews LF (1989). Straight wire: the concept and appliance.

[19] McLaughlin RP, Bennett JC (1989). The transition from standard edgewise to preadjusted appliance systems. J Clin Orthod.

[20] Killiany DM (2002). Root resorption caused by orthodontic treatment: review of literature from 1998 to 2001 for evidence. Prog Orthod.

[21] Leach HA, Ireland AJ, Whaites EJ (2001). Radiographic diagnosis of root resorption in relation to orthodontics. Br Dent J.

[22] Dudic A, Giannopoulou C, Leuzinger M, Kiliaridis S (2009). Detection of apical root resorption after orthodontic treatment by using panoramic radiography and cone-beam computed tomography of super-high resolution. Am J Orthod Dentofacial Orthop.

[23] Linge BO, Linge L (1983). Apical root resorption in upper anterior teeth. Eur J Orthod.

[24] Salehi P, Momeni Danaei S (2007). Comparison of amount of apical root resoprtion in maxillary incisor teeth in 13-18 year old patients with class I, II and III malocclusions, before and after fixed orthodontic treatment. J Mashhad Dent Sch.

[25] Salehi P, Omidkhoda SM, Khojastepour L (2009). Comparative study of maxillary incisors apical root resorption in patients with malocclusion in vertical dimension, before and after fixed orthodontic treatment. J Mash Dent Sch.

[26] Ravanmehr H, Javadin M (2006). Changes in root lengths of maxillary incisors during orthodontic retention period. J Dent, Tehran Uni Med Sci.

[27] Mirabella AD, Artun J (1995). Risk factors for apical root resor-ption of maxillary anterior teeth in adult orthodontic pa-tients. Am J Orthod Dentofacial Orthop.

[28] Artun J, Van 't Hullenaar R, Doppel D, Kuijpers-Jagtman AM (2009). Identification of orthodontic patients at risk of severe apical root resorption. Am J Orthod Dentofacial Orthop.

[29] Mohandesan H, Ravanmehr H, Valaei N (2007). A radiographic analysis of external apical root resorption of maxillary incisors during active orthodontic treatment. Eur J Orthod.

[30] Sameshima GT, Sinclair PM (2001). Predicting and preventing root resorption: Part I Diagnostic factors. Am J Orthod Dentofacial Orthop.

[31] Nigul K, Jagomagi T (2006). Factors related to apical root resorption of maxillary incisors in orthodontic patients. Stomatologija.

[32] DeShields RW (1969). A study of root resorption in treated Class II, Division I malocclusions. Angle Orthod.

[33] VonderAhe G (1973). Postretention status of maxillary incisors with root-end resorption. Angle Orthod.

[34] Taner T, Ciğer S, Sençift Y (1999). Evaluation of apicalrootresorptionfollowingextractiontherapy in subjects with Class I and Class II malocclusions. Eur J Orthod.

